# Concurrent cardiac sarcoidosis and obstructive sleep apnea presenting as arrhythmias

**DOI:** 10.1186/s12890-020-1163-5

**Published:** 2020-05-08

**Authors:** Foteini Malli, Fotini Bardaka, Konstantinos I. Gourgoulianis, Zoe Daniil

**Affiliations:** 1 Respiratory Medicine Department, University of Thessaly School of Medicine, University Hospital of Larissa, Mezourlo (Biopolis), 41110 Larissa, Greece; 2 Faculty of Nursing, University of Thessaly, Larissa, Greece

**Keywords:** Sarcoidosis, Obstructive sleep apnea, Cardiac sarcoidosis, Case report

## Abstract

**Background:**

Cardiac involvement is a rare and potentially fatal presentation of sarcoidosis. Obstructive sleep apnea may complicate sarcoidosis.

**Case presentation:**

We report a case of a sarcoidosis patient with cardiac involvement presenting with ventricular arrhythmias. Besides medical and invasive measures of therapy, the patient failed to respond fully. The patient was subjected to overnight polysomnography and diagnosed with concurrent obstructive sleep apnea syndrome. Following continuous positive airway pressure therapy, we observed a significant improvement of ventricular arrhythmias while methylprednisolone was further tapered.

**Conclusions:**

To our knowledge, this is the first report of cardiac sarcoidosis further implicated by OSAHS and presenting as ventricular arrhythmias that underlies the need for extensive testing in cardiac sarcoidosis in patients not responding to immunosuppressive therapy.

## Background

Sarcoidosis is a rare multisystemic disease that frequently involves various organs and tissues [1]. Cardiac involvement is one of the least common disease presentations but with potentially fatal effects. Obstructive sleep apnea hypopnea syndrome (OSAHS) affects as many as 2–4% of the adult population and presents with signs, symptoms and consequences that result from the derangements associated with the repetitive collapse of the upper airway and include sleep fragmentation, hypercapnia, hypoxemia, fluctuations in intrathoracic pressure, and enhanced sympathetic activity [2]. Cardiac arrhythmias often complicate OSAHS [3].

OSAHS coexistence with sarcoidosis has been previously reported but the exact prevalence is unknown [4, 5]. Here we report for the first time in the literature a case of cardiac sarcoidosis complicated by OSAHS. Our case report underlies the need for extensive testing in cardiac sarcoidosis patients not responding to therapy.

## Case presentation

**Fig. 1:**
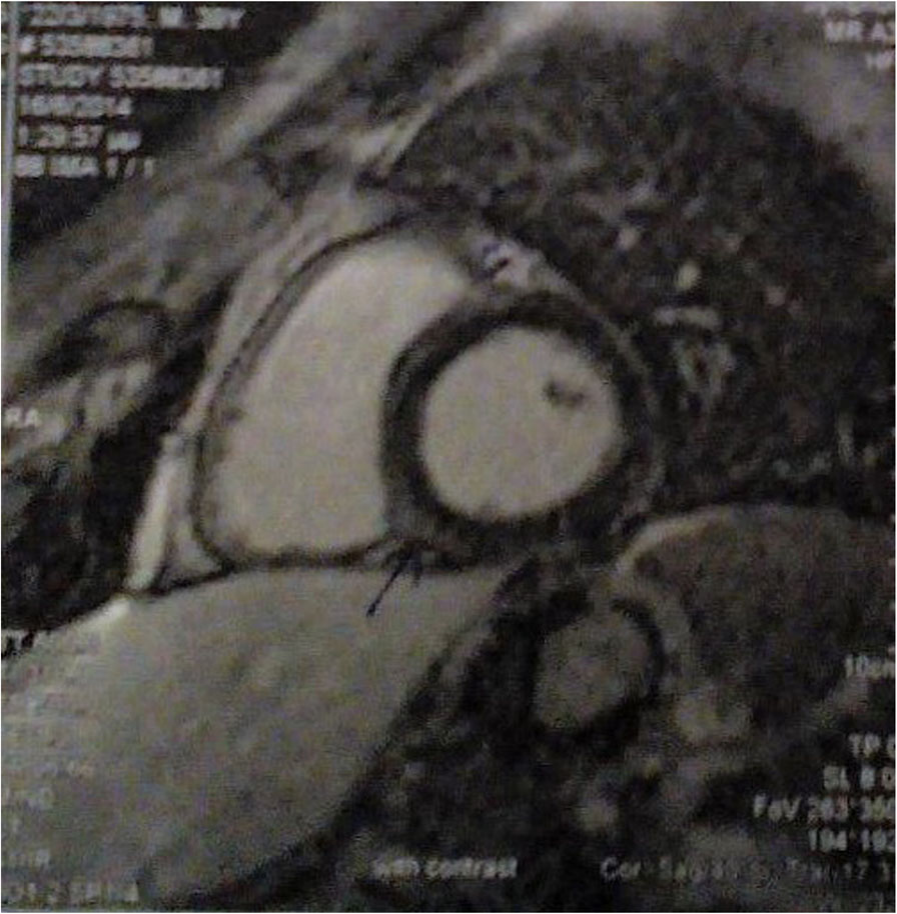
cMRI of our patient showing areas of late gadolinium enhancement consistent with cardiac sarcoidosis

A 41-year-old man with sarcoidosis was referred to our Interstitial Lung Disease Clinic. The patient was investigated 2.5 years ago due to cough. Computed tomography (CT) of the chest revealed enlarged hilar and mediastinal lymph nodes and bronchoalveolar lavage was lymphocytic (lymphocytes 63% of total cells) with a CD4/CD8 ratio of 3.7. Bronchial biopsy revealed non-caseating granulomas. The patient presented 19,804 episodes of non-sustained monomorphic ventricular arrythmias at continuous cardiac monitoring and was subjected to cardiac magnetic resonance imaging (cMRI) that showed areas of late gadolinium enhancement consistent with cardiac sarcoidosis according to published guidelines [1] (Fig. 1). He received corticosteroids for 6 months with clinical and radiological improvement. Additionally, he presented reduced number (17,751) of ventricular arrhythmias when he received 32 mg of methylprednisolone but there was no other continuous cardiac monitoring performed (other than the two measurements reported) at that point of time.

The patient was referred to our department for further evaluation. At presentation he was asymptomatic with hilar and mediastinal lymphadenopathy and was not receiving corticosteroids. His BMI was 29Kg/m^2^. Due to the history of cardiac involvement, he was subjected to continuous cardiac monitoring that revealed multiple (20,729) episodes of non-sustained monomorphic ventricular arrhythmias. He was advised to start corticosteroids (methylprednisolone 32 mg once daily). We observed reduction in the number of ventricular arrhythmias (14,004) when the patient received 28 mg of methylprednisolone. Despite the initial improvement and while on corticosteroid tapering, (methylpredinosolone 24 mg) continuous cardiac monitoring revealed worsening with 17,500 episodes of ventricular arrhythmias. Repeated cMRI revealed stability of findings with no new areas of scarring. He was further subjected to electrophysiological study and ablation was performed. Pacemaker and intracardial defribillator (ICD) was implanted while the patient was started on antiarrhythmic drugs (atenolol). Although these led to improvement of patients’ arrhythmias, progressive tapering was unsuccessful due to worsening of arrhythmias. Positron Emission tomography and Computed Tomography (PET/CT) was performed due to pacemaker implantation (while the patient was receiving immunosuppressive therapy) with normal findings of the heart, suggesting that the patient had no detectable inflammation due to sarcoidosis. PET/CT was performed with proper pre-imaging preparation and the patient was on a carbohydrate free diet for the previous 24 h before the date of the appointment. Additionally, PET scan did not reveal signs of upper airway and peripheral/respiratory muscle involvement. Assessment of other reasons of cardiac arrhythmias revealed symptoms consistent with OSAHS (i.e. snoring) that he reported to be present before sarcoidosis diagnosis and had not worsened while on corticosteroids. His BMI was stable despite steroid therapy. His Malampati score was 2 and Epworth Sleepiness score was 9. The patient was subjected to polysomnography with severe obstructive sleep apnea (AHI = 50.7). Following CPAP therapy, the patient showed significant improvement of ventricular arrhythmias while methylprednisolone was further tapered. Patient compliance to CPAP was high with 6 h and 15 min of cPAP use 99.2% of time. The residual AHI was 2. The patient is currently receiving 8 mg of methylpredinisolone (2.5 years following his referral to our department) while continuous cardiac monitoring revealed reduced (2790) number of ventricular arrhythmias.

## Discussion and conclusions

Cardiac sarcoidosis involves 5% of patients with systemic sarcoidosis while clinically silent sarcoidosis involvement is seen in 25–35% of patients [1]. Ventricular arrhythmias are a common presentation of cardiac involvement with immunosuppression agents, antiarrhythmic drugs, ablation, pacemaker and ICD implantation presenting the available treatment options. OSAHS is a common disorder affecting 2–4% of the adult population [2]. Cardiac arrhythmias are commonly seen in OSAHS patients [3]. Interestingly, patients with sarcoidosis are at higher risk of developing OSAHS possibly due to factors such as muscle involvement, upper airway infiltration and/or steroid use [4]. Turner et al. [5] reported a high frequency of OSAHS in sarcoidosis which was associated with high occurrence of lupus pernio. In our patient PET/CT did not reveal signs of granulomas infiltration of the upper airway or peripheral/respiratory muscle involvement. Additionally, the patient reported stability of symptoms related to OSAHS from the diagnosis of sarcoidosis till the diagnosis of OSAHS while his BMI remained stable despite steroid therapy, suggesting that these factors did not, at least significantly, contribute to the coexistence of the two entities.

In our patient, cMRI prior to CS initiation revealed areas of late gadolinium enhancement consistent with myocardial scarring, while PET/CT during the course of the disease (with the patient receiving immunosuppressive therapy) did not demonstrate areas of active inflammation. Currently, studies have suggested that myocardial scars (identified primarily by cMRI) and not sarcoid inflammation (as suggested by PET/CT) play the dominant role in ventricular arrhythmogenesis [6]. Although available guidelines do not require both cMRI and PET/CT performance for the diagnosis of cardiac sarcoidosis, researchers have suggested that both methods should be used complementary to enable a more accurate and sensitive diagnostic algorithm for cardiac involvement [6].

To our knowledge, this is the first report of cardiac sarcoidosis further implicated by OSAHS. In our case, we assume that the patients’ arrhythmias were probably attributed to cardiac involvement and worsened by coexistent OSAHS. One could argue that we did not perform overnight polysomnography simultaneously with the patients’ diagnosis. Taken the above into consideration, our report strengthens the possible coexistence of the two entities and highlights the need for extensive testing in cardiac sarcoidosis in patients not responding to immunosuppressive therapy.

## Data Availability

Data sharing is not applicable to this article as no datasets were generated or analyzed during the current study.
